# NOVEL GENE VARIANTS ASSOCIATED WITH HBA1C CHANGES OVER TIME AMONG NONDIABETIC SUBJECTS IN THE LONG LIFE FAMILY STUDY

**DOI:** 10.1093/geroni/igad104.3496

**Published:** 2023-12-21

**Authors:** Siyu Wang, Bharat Thyagarajan, Joseph Lee, Joseph Zmuda, Kaare Christensen, Michael Province, Ping An

**Affiliations:** Washington University in St. Louis, St. Louis, Missouri, United States; University of Minnesota, Minneapolis, Minnesota, United States; Columbia University, New York City, New York, United States; University of Pittsburgh, Pittsburgh, Pennsylvania, United States; University of Southern Denmark, Odense, Syddanmark, Denmark; Washington University School of Medicine, St. Louis, Missouri, United States; Washington University in St. Louis, St. Louis, Missouri, United States

## Abstract

Longitudinal changes in HbA1c (ΔHbA1c) are associated with insulin resistance, aging, cognition, and mortality. To identify novel gene variants underlying ΔHbA1c, we conducted linkage-guided sequence analysis on 17p12 (LODs=3.59) in the Long Life Family Study, a study with familial clustering of exceptional longevity in the US and Denmark. Subjects with clinical diagnosis of diabetes or diabetes treatment and undiagnosed diabetes cases whose fasting glucose ≥ 126 mg/dl or HbA1c ≥ 6.5% were excluded. ΔHbA1c, collected from two exams 7 years apart, was derived using growth curve modeling, adjusting for age, sex, BMI, smoking, field centers and PCs, and was blom-transformed to approximate normality. We identified a significant variant under the linkage peak (ARHGAP44 rs56340929, p=1.77E-06, MAF=6%, accounting for linkage=26.5%). Taking advantage of our currently available RNAseq data, we found a significant association between quantification of ARHGAP44 RNA transcript and adjusted ΔHbA1c using 16 linkage enriched families (n=176, β = -0.24, SE=0.09, p=0.01). We also assessed currently available lipidomics data (188 metabolites, 13 compound classes) and found phosphatidylcholine (p=0.025) and lysophosphatidylcholine (p=0.02) were marginally associated with adjusted ΔHbA1c. ARHGAP44 is reportedly associated with glycemic traits and is mainly expressed in the brain. Further complete omics-data analyses in the LLFS and a replication study using the Framingham Offspring Study are underway.

